# Morphology of nuclear transcription

**DOI:** 10.1007/s00418-016-1412-0

**Published:** 2016-02-04

**Authors:** Klara Weipoltshammer, Christian Schöfer

**Affiliations:** Department for Cell and Developmental Biology, Medical University of Vienna, Schwarzspanierstr. 17, 1090 Vienna, Austria

**Keywords:** Nuclear architecture, Nucleoli, Transcription factory, Chromatin, Loop formation, Epigenetic gene regulation

## Abstract

Gene expression control is a fundamental determinant of cellular life with transcription being the most important step. The spatial nuclear arrangement of the transcription process driven by RNA polymerases II and III is nonrandomly organized in foci, which is believed to add another regulatory layer on gene expression control. RNA polymerase I transcription takes place within a specialized organelle, the nucleolus. Transcription of ribosomal RNA directly responds to metabolic requirements, which in turn is reflected in the architecture of nucleoli. It differs from that of the other polymerases with respect to the gene template organization, transcription rate, and epigenetic expression control, whereas other features are shared like the formation of DNA loops bringing genes and components of the transcription machinery in close proximity. In recent years, significant advances have been made in the understanding of the structural prerequisites of nuclear transcription, of the arrangement in the nuclear volume, and of the dynamics of these entities. Here, we compare ribosomal RNA and mRNA transcription side by side and review the current understanding focusing on structural aspects of transcription foci, of their constituents, and of the dynamical behavior of these components with respect to foci formation, disassembly, and cell cycle.

## Introduction

Proper regulation of gene expression is of utmost importance to ensure maintenance of cell integrity, coordinated cellular differentiation, and adequate responses to external and internal cues.

Nuclear transcription is exerted by three RNA polymerases, RNA polymerase I (Pol I) exclusively dedicated to transcription of ribosomal RNA (exception: 5S rRNA), RNA polymerase II (Pol II) transcribes mRNA and noncoding RNAs, and RNA polymerase III (Pol III) produces tRNAs and 5S rRNA.

With the introduction of labeled nucleosides that become incorporated into nascent RNA, it was realized that nuclear transcription does not occur randomly distributed throughout the nuclear volume but rather is found in discrete regions within the nuclear space (Haeusler and Engelke 2006; Jackson et al. 1993; Wansink et al. 1993). These foci were shown to colocalize with the active forms of the polymerases as well as with actively transcribed genes, thus establishing these foci as important entities in the process of gene expression (Cisse et al. 2013; Ghamari et al. 2013; Iborra et al. 1996; Schoenfelder et al. 2010; Verschure et al. 1999) where most of nuclear transcription takes place (Buckley and Lis 2014; Deng et al. 2013; Edelman and Fraser 2012). These foci were named transcription factories (TFs) (Iborra et al. 1996). Existence of compartmentalized transcription sites within nuclei is considered necessary to ensure efficient RNA production by increasing temporal and spatial availability of molecules involved in gene expression control. It was shown that genes tend to share transcription factories, that co-regulated genes often occupy one and the same transcription factory, and that specialized transcription factories exist for certain genes (Denholtz et al. 2013; Li et al. 2012, 2013; Papantonis et al. 2012; Park et al. 2014; Schoenfelder et al. 2010). However, the concept of transcription factories is not universally accepted, and concerns about the formation of transcription factories and about functional consequences were raised [for a review, see (Sutherland and Bickmore 2009)].

Transcription by Pol I is often understood as paradigmatic for localized nuclear transcription and sometimes embraced by the term transcription factory. Nevertheless, there exist clear differences between Pol I and Pol II transcription, e.g., the nucleolus is the site of the by far highest transcription rates in the nucleus, the nucleolus-specific ribosomal DNA (rDNA) is present in a unique structure, rDNA is the only target of Pol I, and transcription is spatially confined to a conspicuous nuclear organelle, the nucleolus [for reviews, see, e.g., (Farley et al. 2015; Raska et al. 2006)].

In this review, we compare nucleoli with transcription factories in the nucleoplasm, in particular Pol II factories, by highlighting commonalities and differences between these two entities with a focus on morphological considerations in mammalian cells.

## Morphology of transcription sites

### Transcription factories

**Fig. 1:**
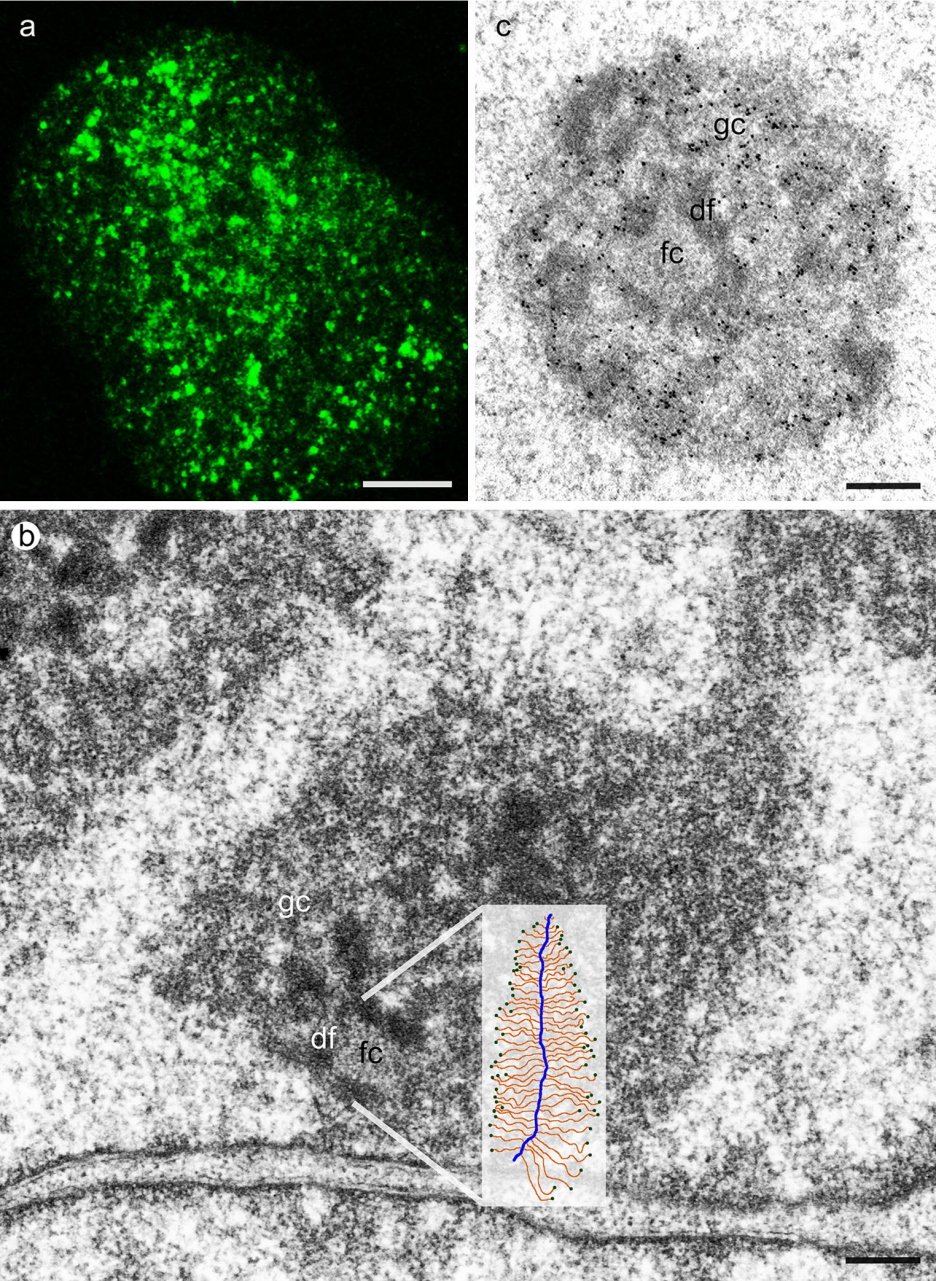
**a** BrU incorporation to visualize nascent transcripts, HeLa cell, confocal image (projection) *Bar* 5 µm **b** Nucleolus of HeLa cell, sketch of Christmas tree in relation to the fibrillar complex where transcription takes place (*inset*), TEM *Bar* 1 µm **c** In situ hybridization to detect rRNA which is present in df and gc, HeLa cell, TEM, *Bar* 1 µm, *fc* fibrillar center, *df* dense fibrillar component, *gc* granular component

Unlike nucleoli, TFs cannot be morphologically identified in the microscope. Initially, they were detected by tagging nascent transcripts (Fig. 1a) via incorporation of labeled nucleotides (Jackson et al. 1993). Later, immunohistochemical detection was introduced because it is more versatile (e.g., antibodies against the elongation-specific Ser2P RNAP II). Pol II subunit fusion proteins have recently been used to visualize transcription factories and allow to study transcription factory dynamics in real time (Cisse et al. 2013). As the average diameter of transcription factories is about 87 nm [for comparison the fibrillar center (FC), a functional analogue in human nucleoli, and measures on average about 500 nm], attempts were made to localize transcription factories at the ultrastructural level. Previous EM studies found that the perichromatin compartment, i.e., the area surrounding dense chromatin, is the site of Pol II transcription (Fakan 1976, 1994; Fakan and van Driel 2007). Detection of transcription factories in conventional TEM preparations using immunogold-labeling (Iborra et al. 1996; Wansink et al. 1996) shows labeled entities outside dense chromatin touching the chromatin surface. These findings were corroborated using a correlative light microscopy/EFTEM approach on erythroid cells where it was shown that transcription factories consist of a protein-rich core surrounded by nucleosomal chromatin (Eskiw and Fraser 2011). Furthermore, in this study, it was shown that during cellular differentiation, the transcription factories were larger than those of cancer cells described previously which is consistent with the hypothesis that coregulated genes tend to occupy specialized transcription factories. Another question concerns the localization of promoters, gene bodies, and nascent transcripts in relation to transcription factories. The kinase CDK9 is responsible for the progress of transcriptional elongation by imparting Ser2P modification of Pol II. In a live-cell approach (Ghamari et al. 2013), it was used to correlate with Ser2P (transcription elongation) and Ser5P (transcription initiation) antibodies. It was shown that Ser5P always colocalized with CDK9, whereas Ser2P extended away from transcription factories, suggesting that initiation and elongation takes place in different compartments (Ghamari et al. 2013).

### Nucleoli

The nucleolus is the morphological correlate of ribosome biogenesis. It is subdivided into three compartments that can be distinguished based on morphological, molecular as well as functional criteria. Electron microscopic images (Fig. 1b) show that two fibrillar components can be distinguished with the FC being less electron dense than the surrounding dense fibrillar component (DF). Both structures lie embedded in the granular component (GC). rRNA transcription is associated with the fibrillar components, whereas later steps of rRNA processing and assembly of ribosomal subunits take place in the GC (Fig. 1c). Nucleolar transcription takes place at the interface between FC und DF (Hozak et al. 1994; Mosgoeller et al. 2001), and occurs there in foci (Mosgoeller et al. 1998). Consistent with these observations, Pol I and the important transcriptional cofactor UBF (upstream binding factor) are most abundant in FCs and occur at lower intensities in DF. rRNA can be found in DF and GC where coordinated processing of the 45S pre-rRNA takes place. The precise topology of active TUs and flanking IGS is still not known in detail. Previously, it was shown with conventional wide-field microscopy that TU and IGS are colocalized (Wachtler et al. 1991), and recently in a 3C study, a modified model was proposed suggesting a core–helix rDNA structure (Denissov et al. 2011).

In HeLa cells, it was estimated that one FC houses about 500 molecules of Pol I, and it is estimated that around 100 Pol I molecules are active on a single TU. Approximately four TUs are transcribed per FC/DF interphase, and because a HeLa cell contains approximately 30 FC foci, it was calculated that there exist around 100–120 active TUs per HeLa cell (Dundr et al. 2002a; Haaf et al. 1991; Jackson et al. 1998).

The transcriptionally inactive rDNA genes are predominately localized at the periphery of nucleoli where they are often found in the vicinity of larger heterochromatin aggregations called NADs (nucleolus-associated domains). In cells with low rRNA transcription rate, small foci of rDNA (satellite nucleoli) can sometimes be seen lying in the nucleoplasm.

## Molecular constituents of nuclear transcription foci

### Chromatin

#### Transcription factories

The transcribed sequences differ substantially between Pol I and Pol II transcription sites. The sequences that attach to transcription factories are typically the promoters of actively transcribed or poised genes, which are accessible for Pol II molecules. Sequences that can be found in one transcription factory can come either from neighboring or distant loci of the same chromosome territory (CT) or from separate CTs. In the latter case, they can form long-range loops as often observed in coregulated loci during cell differentiation processes (Park et al. 2014). This spatial organization is understood to be pivotal for transcription linking chromatin architecture to coordinated gene expression (Kagey et al. 2010; Zhang et al. 2013). Prime examples for such long-range loop associations of induced genes forming a transcription factory are the differentiation-related activation of globin genes. The highly expressed ß-like globin gene *Hbb*-*b1* in murine erythroid cells shows significant colocalization with transcription factories (Osborne et al. 2004). In addition, it was demonstrated that the locus control region (LCR) which is needed for efficient transcription of ß-globin genes is crucial for the association of ß-globin genes with active forms of Pol II (Ragoczy et al. 2006). Subsequently, the association of murine globin genes was confirmed in a genome-wide study, and furthermore, it was shown that the globin-specific transcription factor *Klf1* mediates the co-association of *Klf1*-regulated genes which are found in specialized transcription factories (Schoenfelder et al. 2010). Recently, differentiation-related long-range interactions at transcription factories were also reported for the immunoglobulin genes. This association of the *Ig* genes is hypothesized to facilitate the somatic recombination process (Verma-Gaur et al. 2012). In a comprehensive study on *Ig* genes during B cell development, a pronounced colocalization of the *Ig* genes was found albeit they reside on different chromosomes (Park et al. 2014).

As shown in these and other examples (e.g., (Ho et al. 2013)), loop configuration brings together genes in *cis* or *trans* at transcription factories with the latter often being co-regulated genes involved in differentiation processes (Rao et al.  2014). Indeed, a study using genome-wide chromatin interaction analysis with paired-end tag sequencing (ChIA-PET) found enriched promoter–promoter interactions at transcription factories (Li et al. 2012). The authors further indicate significant enrichment of enhancer–promoter interactions for cell-type-specific transcription and put forward the notion that the observed interactions serve as structural framework for transcription regulation.

Further entities that have shown to be crucial for interactions between promoters and enhancers are insulators and chromatin remodeling complexes. Insulators are crucial for bringing promoters and enhancers in close proximity forming an active chromatin hub (ACH) (de Laat and Grosveld 2003). For instance, the insulator protein CTCF has been found implicated in the localization of active genes to transcription factories. This translocation is dependent on the activity of the proteins of the Trithorax group, which represent euchromatin-promoting factors. These observations strengthen the importance of insulators and chromatin remodelers for loop formation (Li et al. 2013). Cohesin has been shown to bind to similar sites in the genome as CTCF (Wendt et al. 2008), and it has been demonstrated that cohesin is loaded at promoters by transcription factors which aid in establishing loops by interaction with enhancer elements (Kagey et al. 2010). Indeed, a recent study showed that the majority (>85 %) of loops are anchored by CTCF and cohesion underlining the importance of the two insulators for loop formation (Rao et al. 2014).

It has been postulated that other mechanisms than direct insulator-mediated promoter–enhancer interactions might be implicated in loop formation. In the “active nuclear compartment” model, loop formation is the result of the three-dimensional folding of chromatin (maintained by insulators), which place regulatory elements in the same nuclear compartment (Gavrilov et al. 2013; Kosak and Groudine 2004).

Other factors were also implicated in loop formation. Using genome-wide ChIP-Seq, it was found that the chromatin remodeling SWI/SNF complex associates with active Pol I and Pol III sequences indicating participation in loop formation (Euskirchen et al. 2011).

Consistent with its role in gene expression, chromatin associated with transcription factories is enriched in histone marks for active chromatin such as H3K4me3 (Barski et al. 2007; Li et al. 2012). Importantly, the authors further demonstrate interactions between promoters of different genes. Weak promoters were significantly more active when in the vicinity of a strong promoter, which suggests complex combinatorial interactions at transcription factories.

In another study, it was found that retroelements (short interspersed elements (SINEs)) near the promoters of inducible genes were frequently found in transcription factories upon transcriptional induction and in concert with transcription factor recruitment help to localize the respective genes to transcription factories (Crepaldi et al. 2013).

#### Nucleoli

The rRNA is transcribed from rDNA, which is present in a unique conformation in the genome. Individual genes are arranged in a repetitive head-to-tail orientation. The transcribed genes (transcription units; TU) are separated by intergenic spacer sequences (IGS). Several tens of such repeats form a nucleolar organizer region (NOR) which, in humans, lies embedded in extended heterochromatic areas on the short arms of the five acrocentric chromosomes 13, 14, 15, 21, and 22, forming a cytogenetically detectable secondary constriction. It is estimated that on average, 400 TUs exist in the diploid human genome. Interestingly, the number of TUs in the different NORs as well as in different individuals is variable, and it has been shown that not all NORs necessarily participate in transcription (Roussel et al. 1993; Wachtler et al. 1986). The number of NORs participating in nucleolar transcription is under epigenetic control (McStay and Grummt 2008; Schlesinger et al. 2009; Shiao et al. 2011).

**Fig. 2:**
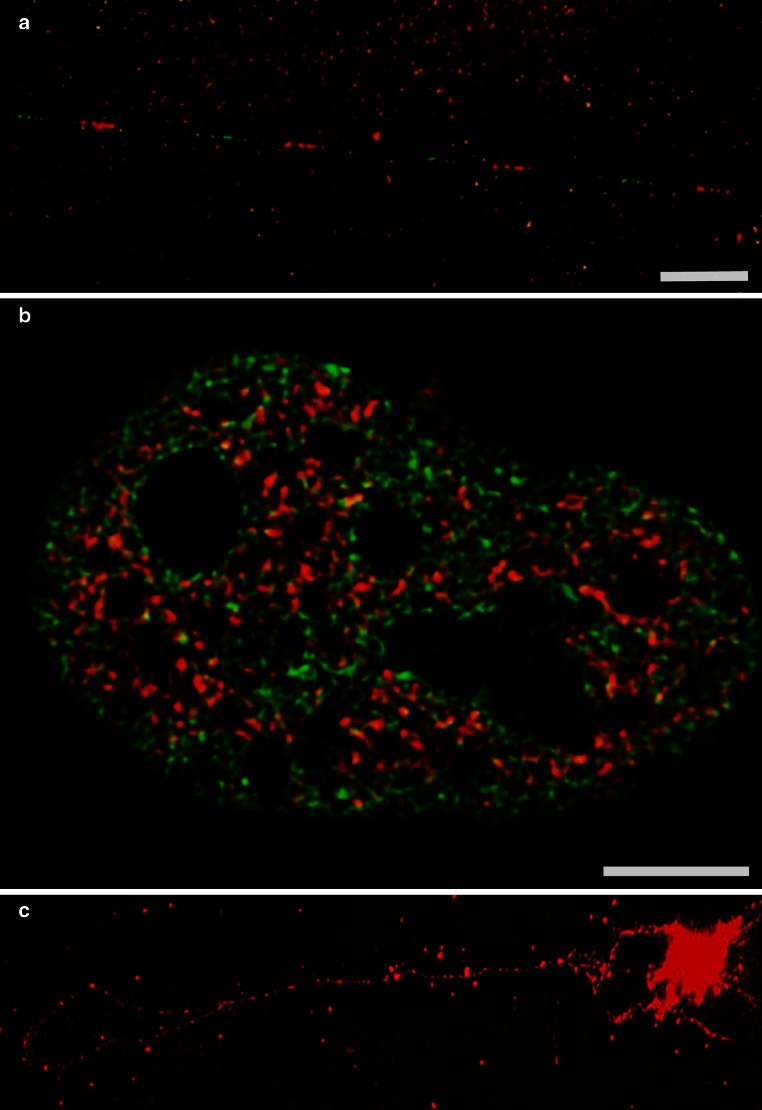
**a** Detection of a fragment of the transcription unit (*red*) and of the intergenic spacer (*green*) of rDNA, FISH on stretched DNA fibers, nuclear halo preparation, *Bar* 5 µm, **b** HeLa cell expressing histones H2B (*green*) and histone H3 variant H3.3 (*red*) which has been associated with transcriptional activity (Ahmad and Henikoff 2002), note that nucleoli are largely devoid of signal, structured illumination imaging, *Bar* 5 µm, **c** FISH with a probe covering the entire rDNA repeat showing an extracted rDNA loop, nuclear halo preparation

The exact number and degree of variation in composition of individual repeats is largely unknown, mostly because molecular methods to analyze single repetitive elements in a genomic context have been missing. For HeLa cells, it was calculated that about 120 TUs are actively transcribed at a given time which means much less than 50 % of TUs are active (Jackson et al. 1998). It was however shown that the number of rDNA repeats and the number of those repeats engaged in rRNA transcription vary individually. By DNA fiber preparations (Fig. 2a), it could be demonstrated that repeat lengths also show differences (Schofer et al. 1998), and that some arrays of repeats display aberrant palindromic arrangements incompatible with transcriptional activity (Caburet et al. 2005). In addition, single repeats differ in their epigenetic states, and inactive repeats were found interspersed with active ones (Zillner et al. 2015). The rDNA tandem array arrangement possesses a high degree of genomic instability, which can result in an age-dependent reduction in the number of TUs as a consequence of damaging environmental influences (Gibbons et al. 2015). In addition, NORs were identified as hot spots of recombination with elevated levels of rDNA rearrangements in solid cancer tissues (Stults et al. 2009).

The epigenetic state of nucleolar chromatin is crucial for the control of gene expression in response to metabolic demands [for a review, see, e.g., (Hamperl et al. 2013)]. The rDNA gene body, in particular the core promoter and the upstream control element of rDNA, is CpG-rich, and thus, DNA methylation is an important regulator of rRNA gene expression (Grummt and Pikaard 2003). The NuRD complex (nucleosome remodeling and histone deacetylase), which is involved in gene silencing in the nucleoplasm, acts as a positive regulator of rRNA transcription by keeping rDNA promoters hypomethylated and thus poised for transcription (Brown and Szyf 2008; Xie et al. 2012). Hypermethylation of rDNA is associated with Alzheimer (Pietrzak et al. 2011), aging (Oakes et al. 2003) and premature aging in Werner syndrome patients (Machwe et al. 2000). In cancer tissue, rDNA methylation levels are dysregulated compared to normal tissue (Abranches et al. 1998; Bacalini et al. 2014; Belin et al. 2009; Uemura et al. 2012; Yan et al. 2000). Zillner et al. (2015) have recently found that both hypo- and hypermethylated repeats coexist in individual NORs of normal and cancer cells.

The three DNA methyltransferases DNMT1, DNMT3a, and DNMT3b play different roles in rRNA transcription. All three enzymes were found at promoters of inactive rDNA genes. Specific roles were identified for DNMT1 and DNMT3B for differential regulation of rRNA expression from methylated and unmethylated rDNA promoters. Furthermore, DNMT1 cooperates with HDAC2 linking DNA and histone methylation in order to repress rRNA transcription (Majumder et al. 2006). Cells lacking DNMT1 and DNMT3b display disruption of nucleolar morphology and dysregulated rDNA gene recruitment (Espada et al. 2007; Gagnon-Kugler et al. 2009).

In addition, noncoding RNAs also influence the rDNA methylation state. It has been demonstrated that an RNA complementary to the rDNA promoter forms a DNA:RNA triplex, thereby creating a target for DNA methyltransferase DNMT3b, thus inducing de novo methylation of CpG islands (Schmitz et al. 2010).

Histone modification is another process by which rRNA transcription can be regulated [for a review, see (McStay and Grummt 2008). Previous studies have revealed two distinct states of rDNA by psoralen cross-linking methods (Conconi et al. 1989). In one conformation, chromatin consists of nucleosomes that are inaccessible for psoralen, whereas the second state is largely histone-free and accessible for cross-linking. It is now believed that rRNA genes exist in at least three chromatin states. State one consists of silenced chromatin composed of regular nucleosomal architecture in a closed configuration displaying silencing marks like promoter hypermethylation or increased trimethylation of H3K9. Association of rDNA promoters with the nucleolar remodeling complex NoRC maintains the silenced state (Santoro et al. 2002). Transcriptionally active TUs are present in open chromatin configuration characterized by activating marks like promoter hypomethylation and histone dimethylation of H3K4. The active genes contain all necessary components of the transcript machinery. The third part of rDNA is also present in an open configuration with unmethylated promoters but contains bivalent (repressive and active) histone modification marks and is associated with the nucleosome remodeling and deacetylation complex (NuRD). The bivalent nature of these epigenetic marks together with the fact that the promoters are occupied by part of the transcription machinery (but without Pol I) indicates that these rDNA genes are present in a poised state (Xie et al. 2012). The authors hypothesized that the switch from poised to active state is mediated by the chromatin remodeling factor Cockayne syndrome B (CSB).

The precise nature of the open chromatin configuration of the active rDNA gene body is under debate [see, e.g., (Jones et al. 2007; Merz et al. 2008)]. The initial psoralen cross-linking data suggested that active TUs are nucleosome-free which is in line with electron micrographs of Miller spreadings. It was believed that the nucleosome-free arrangement ensures the high Pol I occupancy and elongation rates. However, using the chromatin endogenous cleavage (ChEC) method, it was found that there exist few nucleosomes in the promoter region of active TUs, and that their position and epigenetic modifications differ in active and inactive rDNA genes (Cong et al. 2013; Langst et al. 1998; Li et al. 2006; Majumder et al. 2010).

The nucleosome-depleted gene body and the high occupancy with Pol I molecules seem to argue against a canonical nucleosomal arrangement (Fig. 2b). It has also been put forward that alternate states of nucleosomes might exist in the TU such as the lexosome (Prior et al. 1983) for a review, see (Lavelle and Prunell 2007) that would evade psoralen cross-linking and allow for Pol I progression.

More recently, a study in yeast indicated that Pol I-transcribed nucleosomal rDNA is composed of “dynamic chromatin” consisting of noncanonical nucleosomes (Jones et al. 2007). In another study, it was found that the TU is depleted of histones relative to the IGS (Zentner and Henikoff 2013) and yet another study showed three different nucleosomal architectures of active rDNA repeats in yeast (Johnson et al. 2013).

An intriguing question is how nucleosome depletion is brought about and maintained. One of the many nucleolar functions of the upstream binding factor 1 (UBF1) is to decondense rDNA chromatin (Chen et al. 2004). Indeed, an inverse correlation for rDNA occupation was found between histones and UBF in human cells (Gagnon-Kugler et al. 2009). UBF binds to the entire rDNA gene body and is crucial for forming the enhancesome which brings together promoter and enhancer sequences in a loop formation (Bazett-Jones et al. 1994) functionally resembling and probably replacing nucleosomes. Also, the chromatin remodeling activity of the transcription factor TTF-1 has been implicated in establishing spatial conformation of rDNA by forming a loop which brings together 3′ and 5′ ends of TUs mediated by TTF-1, thus facilitating transcription (Grummt and Langst 2013).

The FACT complex (facilitator of chromatin transcription) which specifically interacts with H2A–H2B histone dimers has been shown to reorganize, evict, or displace nucleotides in order to facilitate the elongation process (Formosa 2012). Recently, an rDNA-specific histone modification mark, glutamine methylation of H2A, was identified, which prevents FACT binding to H2A, and the authors suggested that thus the re-deposition of histones in the gene body by FACT is decreased (Tessarz et al. 2014).

**Fig. 3:**
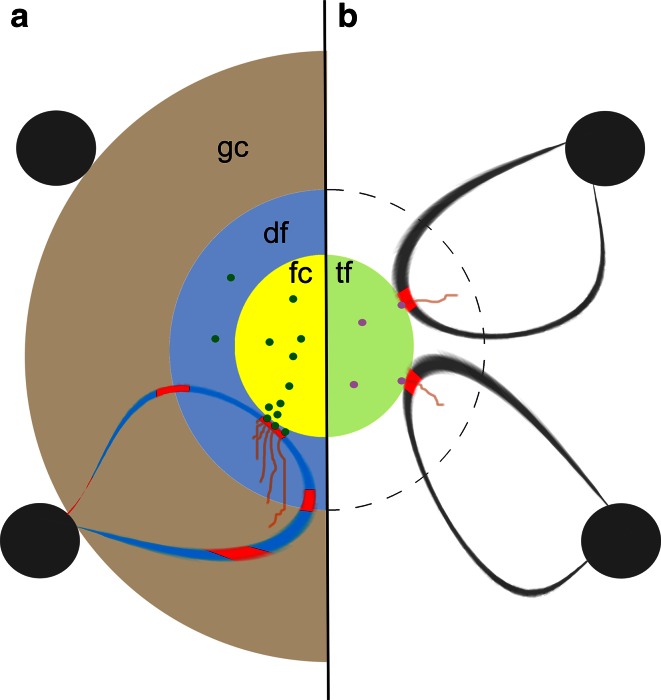
Sketch to compare morphology of transcription **a** in nucleoli and **b** in transcription factories. The transcription factory core (tf) is functionally related to the fibrillar center (fc), and transcription takes place at the surface of both entities. Active genes come into contact with the polymerases by chromatin loop formation out of silenced chromatin (*gray dots*). In nucleoli, nascent transcripts (*brown*) are predominately found in the dense fibrillar component (df) where RNA processing commences. A similar zone can be postulated for the transcription factory, Pol I…*green dots*, Pol II *pink dots*, *gc* granular component

In summary, the chromatin and DNA constituents vary significantly between transcription factories and nucleoli. Basic principles of gene expression such as loop formation (Figs. 2c, 3) apply in both cases, although a more complex DNA arrangement was postulated for active rDNA (Denissov et al. 2011).

### Proteins

#### Transcription factories

Electron spectroscopic imaging of transcription factories suggested a protein mass of 10 MDa (Eskiw et al. 2008). Recently, extraction of all three polymerase complexes became possible, and mass spectrometry revealed several hundreds of protein in each complex (Melnik et al. 2011). In Pol II complexes, Pol II subunits were identified as expected as well as transcription factors, specific regulators such as CTCF, RNA processing factors, and structural proteins such as actin and lamins A/C and B.

#### Nucleoli

In isolated nucleoli analyzed by mass spectroscopy between 700 and 1400 proteins were detected (Ahmad et al. 2009; Andersen et al. 2005). In spite of the high number of proteins, the density of nucleoli has been found only about twice that of the nucleoplasm likely reflecting the dynamics of nuclear components and also functions of nucleoli apart from ribosome biogenesis (Boisvert et al. 2007).

Some nucleolar proteins are involved in several different processes. One multifunctional protein is nucleolin [for reviews, see (Durut and Saez-Vasquez 2015; Mongelard and Bouvet 2007)]. Nucleolin can bind DNA and RNA and is involved in rRNA transcription, processing and ribosomal transport as well as in other nonribosomal functions. Nulceolin has been proposed to be a nucleolar matrix-binding protein in plants (Minguez and Moreno Diaz de la Espina 1996). In the nucleolus, it is found at the interface between FC and DF, in the DF and in rDNA gene bodies consistent with its role in Pol I gene expression. Nucleolin has also been shown to act as histone chaperone binding H2A–H2B dimers and consequently as a histone remodeler. In this respect, nucleolin functionally resembles the nucleoplasmic FACT complex, thus facilitating Pol I transcriptional elongation (Angelov et al. 2006). Furthermore, nucleolin is implicated in preventing binding of the repressive chromatin complex NoRC to active rDNA repeats. Nucleolin possess helicase activity and thus may be involved in rDNA replication. At the same time, nucleolin is involved in the first rRNA processing steps through interaction with U3snoRNP. Nucleolin is thus one of the major nucleolar proteins implicated in regulation of ribosomal biogenesis at different levels.

Another multifunctional nucleolar protein is the methyltransferase fibrillarin [for a recent review, see (Rodriguez-Corona et al. 2015)]. In nucleoli, fibrillarin is localized in the same compartments as nucleolin, i.e., in the FC/DF transition zone and in the DF. Fibrillarin is involved in different rRNA modification processes such as pre-rRNA cleavage, methylation, and ribosome assembly. Recently, fibrillarin was also shown to be the methyltransferase responsible for active Pol I-specific histone glutamine methylation of H2AQ104, which binds FACT (Tessarz et al. 2014). Fibrillarin is a core protein of the U3snoRNP (Baserga et al. 1991); thus, it can be speculated that interaction of fibrillarin and nucleolin might be involved in histone modification and remodeling of active rDNA chromatin.

### RNA polymerases

#### Transcription factories

One main advantage of compartmentalized gene expression lies in the accumulation of relevant transcription factors and in particular of RNA polymerases. Transcription factories are routinely detected in fixed cells with antibodies against Pol II which is phosphorylated at serine2 (Ser2P) in the C-terminal domain (CTD) of RPB1, the largest subunit of Pol II. Different phosphorylation patterns at the CTD are thought to exert different functions [for a review, see (Phatnani and Greenleaf 2006)], and Ser2P-Pol II is engaged with transcriptional elongation, whereas Ser5P and Ser7P are involved in initiation prior to actual transcription (Spilianakis et al. 2003). However, recently several acetylations and methylations of lysine residues were identified in the CTD of RPB1 suggestive of a complex fine-tuning network for initiation and elongation (Dias et al. 2015).

In transcription factories, it was estimated that on average, eight active Pol II molecules are available for transcription initiation and elongation.

For the initiation of efficient Pol II transcription, the enzyme forms an association with five transcription factors at promoter DNA, constituting the preinitiation complex (PIC) [for a review, see (Kornberg 2007)], a process which has recently been studied in real time (Fazal et al. 2015).

#### Nucleoli

In the nucleolus, the regulation of rRNA transcription is tightly coupled to metabolic demands of the cell. The minimal essential Pol I transcription machinery consists of Pol I, SL-1, and UBF [reviewed in (Russell and Zomerdijk 2006); for a review on evolutionary conservation of polymerase subunits, see (Viktorovskaya and Schneider 2015)]. In contrast to Pol II where modification of CTD is essential for transcriptional initiation, elongation and termination in rRNA transcription is dependent on above-mentioned auxiliary factors. These factors are important to guide Pol I and to stabilize Pol I at promoters and are hyperphosphorylated when active. UBF binds directly to DNA and is thought to alter rDNA structure such that promoter sequences and the upstream control element are brought together (Putnam et al. 1994).

### RNA

#### Transcription factories

It is increasingly recognized that RNAs, in particular noncoding RNAs, play important roles as regulators of gene expression but also as determinants for structural components of the transcription machinery. Recently, it became possible to isolate transcription factories and to analyze their RNA content (Caudron-Herger et al. 2015a). It was found that transcription factories contain the entire nascent transcriptome of the cell including noncoding RNA, enhancer-associated RNA (eRNA), repeat-derived RNAs, and micro-RNA precursors (Caudron-Herger et al. 2015a). The authors speculate that in particular the noncoding RNAs may have an influence in regulatory activities of transcription factories. It remains to be seen if those noncoding RNAs are implicated in the formation of chromatin loops as has been suggested for the RNAi machinery including its RNA component (Lei and Corces 2006).

#### Nucleoli

In the nucleolus, Pol I transcripts derived from promoters located in the IGS region (structurally similar to the Pol I gene promoter) have been shown to be important epigenetic modifiers of gene transcription. It could be demonstrated that they are crucial for NoRC targeting and thus for rDNA silencing (Mayer et al. 2006, 2008). Two long noncoding RNAs (lncRNA) derived from IGS are important for remodeling of nucleolar structure in response to environmental cues (Jacob et al. 2013). In addition to these Pol I-dependent transcripts, Pol II transcription of noncoding RNAs has been identified in the IGS. These transcripts were shown to be essential for rDNA silencing by recruitment of the NoRC complex and mediating histone modification (Bierhoff et al. 2014). Protein components of the RNAi machinery were shown to alter nucleolar structure in *Drosophila* (Peng and Karpen 2007), and in plants, RNAi-mediated silencing of rRNA transcription has been reported (Preuss et al. 2008). Very recently, noncoding RNAs from transposable elements have been found to be implicated in maintenance of nucleolar structure (Caudron-Herger et al. 2015b). In this study, it was described that *alu*RNAs derived from Pol II transcription modulate nucleolar structure by interaction with nucleolar proteins nucleolin and nucleophosmin. This establishes a link between Pol I and Pol II transcription and may explain the previously observed phenomenon that inhibition of Pol II transcription leads to nucleolar rearrangement.

## Dynamics of transcription sites

### Dynamics of the constituents

#### Transcription factories

Previous studies demonstrated the existence of two different pools of nuclear Pol II, one pool (~75 % of all Pol II molecules) being mobile and the other (25 %) being transiently immobile (Kimura et al. 2002). By extracting the soluble fraction, it was confirmed that the nonextractable fraction is the transcriptionally active population of the enzyme, which is concentrated at the surface of transcription factories and immobilized there (Eskiw et al. 2008). It has further been suggested that the DNA is pulled through the polymerase rather than the other way around (Iborra et al. 1996). Life cell imaging of Pol II-dependent transcription applying super-resolution microscopy confirmed the focal distribution of the polymerase seen in fixed cells but led to a surprising picture of the dynamics of this enzyme. It was found that the residency time of RPB1 molecules at transcription foci was surprisingly short and that the foci were transient structures (Cisse et al. 2013). The authors calculated the average lifetime of foci to be about 5.1 s. The short lifetime of transcription factories fits well with the fact that regular transcription of mRNA genes occurs in bursts, i.e., in a succession of on–off states, rather than in a continuous mode. The authors could also demonstrate that upon stimulation of transcription, the lifetime of transcription factories increased significantly. This is consistent with an earlier observation on the highly transcribed globin genes in erythroid cells where the authors found that these genes were active in a near-continuous expression mode, whereas other genes studied displayed marked on–off periods (Osborne et al. 2004). Another interesting observation by (Cisse et al. 2013) made in experiments where Pol II transcription was stalled is that formation of transcription factories precedes transcriptional activation.

#### Nucleoli

Pol I and UBF molecules are predominately present within FCs and to a lesser degree in DF of nucleoli (Mosgoeller et al. 1998). Applying lifetime FRAP imaging and mathematical modeling of GFP-tagged UBF and several GFP-tagged Pol I subunits (RPA), a high turnover rate similar to that of Pol II has been demonstrated (Dundr et al. 2002a, b). The authors estimated the presence of 200–400 [around 500 in HeLa cells (Jackson et al. 1998)] molecules of Pol I per FC and 90–520 molecules of UBF per FC (Dundr et al. 2002b). The kinetics revealed that more than 95 % of Pol I molecules exchanged rapidly, and FRAP recovery rates of Pol I and UBF were shown to be in the range of 30–40 s. Initiation of the Pol I machinery occurs every 1.4 s, and on average, it takes 140 s to transcribe one human TU. Together, these findings on the Pol I machinery demonstrate rapid movements through the nucleolus and lead to the conclusion that the parts of the machinery enter the nucleolus as separate subunits rather than as pre-assembled holoenzyme as previously thought (Dundr et al. 2002a). In addition, a high flux rate of nucleolar proteins was also found using a mass spectrometry-based proteomics approach to compare nucleoli of HeLa cells grown under normal conditions and under Pol I inhibition (Andersen et al. 2005). Furthermore, the authors found that only a subset of proteins are influenced by Pol I inhibition which leads the authors to suggest that nucleolar structure forms not only as result of ongoing rRNA synthesis but that a core of proteins remain in the absence of transcription (Andersen et al. 2005). It can be speculated that the different nonribosomal functions of nucleoli [for a review, see (Boisvert et al. 2007)] play a role in formation of the final nucleolar structure.

Together, these data demonstrate similarities with high mobility of the transcription subunits of both Pol I and Pol II. In terms of the transcription process, a succession from on–off states (e.g., house-keeping genes) to near-continuous (strongly expressed genes during differentiation) to continuous transcription (rDNA) can be observed. Morphologically, alterations occur at very different timescales, seconds in Pol II transcription factories and minutes for nucleoli when, e.g., treated with transcriptional inhibitors or enhancer drugs.

### Formation, maintenance, and disassembly of transcription foci

#### Transcription factories

The number of foci of ongoing Pol II transcription inside nuclei is much lower than the number of genes transcribed. Indeed, it has been shown that TFs contain more than one transcribed gene. This requires chromatin to be organized such that genes can come into contact. Loops established by promoter–enhancer interactions (PEIs) are pivotal, although other interactions such as promoter–promoter, enhancer–enhancer, or insulator–insulator looping occur.

Differing models have been proposed on the mechanisms by which PEIs form in nuclei. It has been shown that a fraction of Pol II is retained in nuclei after extraction procedures implying that they are tethered to an underlying structural support (nucleoskeleton) (Jackson and Cook 1985). Because it was found that the retained fraction is enriched in active Pol II, a model was proposed that the polymerase is fixed and the DNA template is pulled through the transcription complex during transcription. In consequence of this view, transcription factories are in fact pre-assembled organizing structures and chromatin loops are move toward transcription factories. Some findings are difficult to reconcile with this model such as that Ser2P is also found outside transcription factories during elongation inconsistent with fixed polymerase molecules (Ghamari et al. 2013) or the high mobility of Pol II subunits observed in live cells (Cisse et al. 2013).

Nevertheless, the transcription factory model is widely appreciated [e.g., (Chen et al. 2015; Li et al. 2012; Osborne et al. 2004; Rieder et al. 2014; Schoenfelder et al. 2010)] for reviews, see (Edelman and Fraser 2012; Papantonis and Cook 2013). And further support for the pre-assembly model can be found in studies on induction of globin genes and LCR positioning where it was shown that LCR originally is located in the repressive nuclear periphery and translocates into the nuclear interior by loop formation and only then robust transcription is started (Ragoczy et al. 2006; Schubeler et al. 2000) which would localize to transcription factories (Lee et al. 2011; Schoenfelder et al. 2010; Zhou et al. 2006). In a study on the LCR of the gene *hGH*-*N* (human pituitary growth hormone), it was shown that the DNase I-hypersensitive site I (HSI) of the LCR acts as important factor to sustain association of the gene with transcription factories over many years of development (Ho et al. 2013). Conversely, drug-induced transcriptional termination does not lead to disassembly of transcription factories but they remain stable with genes attached (Ghamari et al. 2013; Mitchell and Fraser 2008; Palstra et al. 2008) in spite of the high mobility of transcription factory components observed in live-cell studies (Cisse et al. 2013). In addition, transcription factories can exist in poised states enriched in Ser5P Pol II modification but lacking Ser2P (Ferrai et al. 2010) or can harbor poised genes (Larkin et al. 2012) which in both cases enable quick resumption of gene expression.

The question however whether transcription factories are pre-assembled drivers of nuclear organization or whether the transcriptional compartmentalization is the net result of chromatin folding and consequently of the arrangement of active genes in the nuclear space is debated (Sutherland and Bickmore 2009). The two scenarios are not mutually exclusive, and an attractive hypothesis has been put forward that transcription factories might form as consequence of transcription of highly active genes with strong promoters and that these newly formed transcription factories are able to recruit other genes (Sutherland and Bickmore 2009). It can further be speculated that these newly formed transcription factories may contain Pol II molecules that are fixed in position.

Taking into account the structural basis of nuclear organization, hypotheses have been put forward ranging from existence of a nuclear matrix or nucleoskeleton to stochastic self-assembly of macromolecular complexes driven by biochemical and biophysical forces. Indeed, modeling studies support the possibility of generating clusters (Canals-Hamann et al. 2013), although biophysical forces certainly are important players in the pre-assembly model as well (Brackley et al. 2013).

The onset of mitotic division leads to dispersal of transcription factories. However, it became increasingly recognized that transcriptionally active genes in previous interphase display an epigenetic memory effect of the active state (mitotic bookmarking). This mark is transmitted through mitosis into G1 of daughter nuclei and is thought to ensure robust expression of the same set of genes active in the mother cell. The bookmarking mechanism includes specific histone modifications, histone variant replacement or maintenance of the general transcription factor TIFIID binding to promoters [for review bookmarking, see (Zaidi et al. 2010)]. However, Pol II components detach from promoters in prophase and are sequentially imported into daughter nuclei in G1 (Prasanth et al. 2003).

#### Nucleoli

As mentioned above, in the nucleolus, transcription does also occur in foci at the interface between FC and DF and, importantly, NORs of different chromosomes need to convene to for a proper nucleolus. Usually, NORs are located at the periphery of nucleoli where they are in their silenced state and from there extend loops of rDNA repeats into nucleoli which are transcribed. The convergence of the acrocentric, NOR-bearing human chromosomes takes place in early G1 phase when chromatin is relatively mobile before CTs adopt a stable position (Walter et al. 2003). The mechanisms by which NORs come together and eventually fuse are not understood, and Pol I transcription seems to be dispensable for NOR fusion (Dousset et al. 2000). Distal and proximal NOR-flanking sequences have been found implicated in maintaining nucleolar integrity and, possibly, in NOR fusion (Floutsakou et al. 2013) but not in formation of nucleoli (Grob et al. 2014). NORs need to be competent for fusion which critically depends on UBF occupancy keeping them in a nonheterochromatic state (Grob et al. 2014). Not only NOR fusion is poorly understood also the establishment of the peculiar arrangement of the three nucleolar compartments remains enigmatic. Yet, nucleoli of different cell types often display characteristic morphologies.

The fate of the rDNA transcription machinery during cell cycle is substantially different from Pol II transcription. The major constituents of the Pol I transcription machinery such as Pol I and UBF remain bound to (previously active) NORs throughout mitosis [reviewed in (Hernandez-Verdun 2011)] constituting an effective way of mitotic bookmarking. In prophase, the nucleolar components begin to dissociate, and at late prophase, Pol I transcription is halted by phosphorylation of transcription factors through CDK1-cyclin B kinase. Pol I transcription resumes already in telophase by inhibiting CDK1 and nucleolar assembly commences in early G1 before fusion of NORs. For the formation of proper nucleoli, Pol I transcription is not sufficient and requires components of the rRNA processing machinery that are found in particular nuclear bodies called pre-nucleolar bodies (PNBs) such as snoRNAs, ribosomal proteins, and unprocessed 45S rRNA which was synthesized before transcription termination. Some of the constituents decorate mitotic chromosomes and are being transferred to nucleoli in G1 ensuring the formation of normal nucleoli followed by fusion of nucleoli (Hernandez-Verdun 2011).

**Fig. 4:**
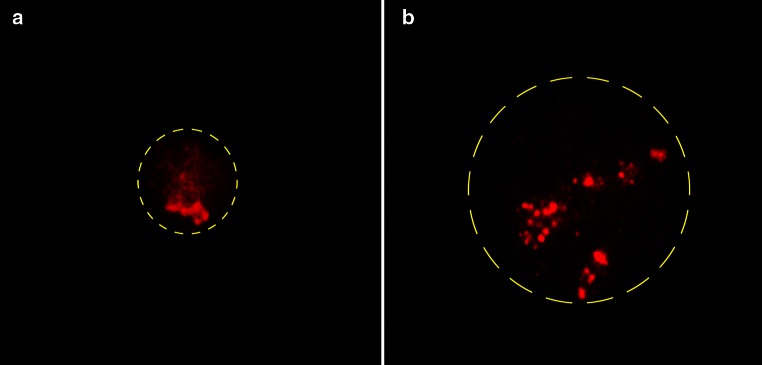
Human peripheral lymphocytes **a** unstimulated and **b** after 72-h stimulation, FISH to detect part of the TU of rDNA showing significant alterations of rDNA arrangement in the course of differentiation, nuclear outline indicated (*yellow*)

Interestingly, the number of nucleoli varies even within one population of a cell line, although there exist less active NORs than the ten NORs in humans (Carpenter et al. 2006). The structure of the nucleolus is continuously present throughout interphase yet it changes its structure dramatically depending on cellular demand of ribosomes. As an example, resting lymphocytes contain nucleoli with maximum of nine FCs, whereas after stimulation the number of FCs increases to about 80 (Haaf et al. 1991) which is accompanied by drastic changes in number and size of nucleoli (Fig. 4) (Wachtler et al. 1982). Interestingly though, it has previously been reported that the number of active rDNA repeats is stable throughout interphase (Conconi et al. 1989). Transcriptional upregulation can also be mediated by epigenetic induction (Stefanovsky and Moss 2008) or by increasing the occupancy of TUs with Pol I molecules. The latter has been shown to be the case in yeast (French et al. 2003) where a strain with reduced number of rDNA repeats compensated this deficiency by higher Pol I occupancy. Indeed, the theoretical minimal occupancy rate in yeast was determined to be 1 Pol I molecule per 35 nt (Tornaletti et al. 1992) which, related to humans, would add up to about 380 molecules per TU instead of the estimated 100 (Haaf et al. 1991), suggesting some upregulation potential for human rRNA transcription. On the other hand, in human cells it was shown that the pool of rDNA genes is not static and that UBF determinates the number of active genes (Sanij et al. 2008).

Genetic engineering approaches have been conducted to gain insight into the requirements for nucleolus formation in human cells. The ectopic insertion of an UBF-binding sequence in a cell line created a “pseudo-NOR,” i.e., secondary constriction at the integration site upon UBF loading (Mais et al. 2005). The insert did not contain the Pol I promoter sequence and did not form functional nucleoli, although Pol I was recruited by UBF. This group went on to produce “neo-NORs” (Grob et al. 2014) which contain fully functional ectopic rDNA repeats which actively produce ribosomes and some of these NORs fuse with endogenous ones, suggesting the existence of a “NOR-territory” (Grob et al. 2014).

The example of the co-localization of neo-NORs and endogenous NORs in one nucleolus is somewhat reminiscent of co-localization of several genes in one Pol II transcription factory. Similar to transcription factories also in the case of nucleoli, a structural fixation of active Pol I molecules in the FC was demonstrated (Dickinson et al. 1990) as well as preferential retention of the TU versus IGS sequences in HeLa cells using extraction protocols (Weipoltshammer et al. 1996). Also in the case of nucleoli, biophysical forces and self-assembly have been postulated as contributing factors for nucleolus formation and maintenance [reviewed in (Hancock 2014; Lam and Trinkle-Mulcahy 2015)].

## Conclusions

RNA polymerase I transcription has often been put forward as paradigmatic for nuclear transcription. Concerning the constituents, Pol I and Pol II transcription are largely different. However, on a functional level, analogies can be found which may rely on general principles of biological organization. Chromatin loop formation is one hallmark that acts as prerequisite of focal transcription in both transcription factories and nucleoli. Another common property is the high mobility of some of the constituents.

The underlying organizing principles of focal transcription and of nuclear organization in general are controversially discussed and range from fixation to an underlying support such as nuclear matrix or nucleoskeleton to self-organization and compartmentalization based on stochastic biochemical and biophysical principles. The methodological armory for studying biophysical forces is restricted, and novel approaches are needed to reveal if one of these possibilities or combinations thereof occur.

In addition to basic consideration on nuclear organization, focal nuclear transcription might have consequences relevant for medicine. In this respect, it was postulated that the proximity of genes present in an open and transcriptionally active state at transcription factories might have potentially detrimental consequences for genome stability and chromosomal translocations ultimately leading to cancer (Osborne 2014), and it seems as if similar conclusions can be drawn for the stability of the rDNA cluster [e.g., (Diesch et al. 2014; Harding et al. 2015)]. Moreover, involvement of Pol I transcription in many disease-related pathways has been demonstrated (Quin et al. 2014). Furthermore, Pol I transcription has become a promising target for therapeutic intervention by the introduction of cancer-specific, Pol I inhibiting small-molecule drugs (Drygin et al. 2011).
